# Prediction of Sub-Monomer A2 Domain Dynamics of the von Willebrand Factor by Machine Learning Algorithm and Coarse-Grained Molecular Dynamics Simulation

**DOI:** 10.1038/s41598-019-44044-2

**Published:** 2019-06-21

**Authors:** Michael J. Morabito, Mustafa Usta, Xuanhong Cheng, Xiaohui F. Zhang, Alparslan Oztekin, Edmund B. Webb

**Affiliations:** 1 Department of Mechanical Engineering and Mechanics, Lehigh University, Bethlehem, PA 18015 United States; 2 G.W. Woodruff School of Mechanical Engineering, Georgia Institute of Technology, Atlanta, GA 30332 United States; 3 Department of Materials Science and Engineering, Lehigh University, Bethlehem, PA 18015 United States; 4 Department of Bioengineering, Lehigh University, Bethlehem, PA 18015 United States

**Keywords:** Computational biophysics, Molecular conformation

## Abstract

We develop a machine learning tool useful for predicting the instantaneous dynamical state of sub-monomer features within long linear polymer chains, as well as extracting the dominant macromolecular motions associated with sub-monomer behaviors of interest. We employ the tool to better understand and predict sub-monomer A2 domain unfolding dynamics occurring amidst the dominant large-scale macromolecular motions of the biopolymer von Willebrand Factor (vWF) immersed in flow. Results of coarse-grained Molecular Dynamics (MD) simulations of non-grafted vWF multimers subject to a shearing flow were used as input variables to a Random Forest Algorithm (RFA). Twenty unique features characterizing macromolecular conformation information of vWF multimers were used for training the RFA. The corresponding responses classify instantaneous A2 domain state as either folded or unfolded, and were directly taken from coarse-grained MD simulations. Three separate RFAs were trained using feature/response data of varying resolution, which provided deep insights into the highly correlated macromolecular dynamics occurring in concert with A2 domain unfolding events. The algorithm is used to analyze results of simulation, but has been developed for use with experimental data as well.

## Introduction

von Willebrand Factor (vWF) is a large multimeric protein found in blood plasma. vWF plays an indispensable role in the blood clotting process by initiation of clot formation that stops bleeding due to vessel hemorrhage^1–4^. vWF is able to sense elevated hydrodynamic force in blood flow at the site of vascular damage and respond by undergoing conformational changes^5–7^. These changes occur on multiple length scales. First, vWF unravels from a compact globule to an unraveled configuration on a macromolecular length scale, after which a second conformational change may take place on a sub-monomer length scale whereby the A2 domain of vWF is able to unfold if hydrodynamic force is sufficient^8,9^.

This study focuses on the A2 domain of the vWF monomer. Single-molecule pulling experiments have demonstrated that the A2 domain unfolds due to internal tensile forces in the range of 7–15 *pN*^10^, where larger unfolding forces were observed for faster pulling rates. Thus, under the action of hydrodynamic forces in shearing flows, it is expected that A2 domains unfold when subject to forces in the range reported from single-molecule pulling experiments. The partial or complete unfolding of the A2 domain is required for molecular scission by the enzyme ADAMTS13^11,12^, because the vWF cleavage site is deeply buried in the folded A2 domain structure and must be revealed by force-induced A2 domain unfolding in order for vWF proteolysis to occur^13,14^. A2 domain cleavage is crucial for preventing excessive thrombus formation. Large hemostatically active vWF multimers must be cleaved to functional lengths upon secretion into the vasculature in order to avoid adverse blood clotting^15–17^.

Besides vWF proteolysis, it has been reported that the A2 also plays an important role in the platelet binding process^18^. Macromolecular unravelling reveals binding sites in the A1 domain for the platelet receptor GpIb*α*, which enables vWF to act as a bridge molecule in the binding of platelets to damaged blood vessels^7^. It has been reported that autoinhibition of vWF-platelet binding exists, and is mediated by a force-sensitive interaction of the A1 and A2 domains^19^. The dissociation of the A1-A2 complex is caused by a stretching force that was found to be coupled with A2 domain unfolding, such that A1-A2 dissociation occurred just before exposure of the cleavage site within the A2 domain. The exact biochemical basis for A1-A2 interaction was recently quantified more precisely^20^. Some of the current authors have advanced simulation data that invoked A2 domain mediation of A3-collagen binding as a mechanism for observing shear-induced binding trends that are in agreement with experiment^21^. While A2 domain mediation of A3-collagen binding has not been confirmed or refuted by experiment, data that have been advanced clarifying A2 mediation of A1-platelet binding were obtained from a number of different experimental methods. It is clear, then, that A2 domain distortion and eventual unfolding are behaviors that are critical to healthy vWF functionality.

Hydrodynamic force induced A2 domain unfolding is an important aspect of vWF functionality. Naturally, the ability to predict A2 domain dynamics would open the door to many possible scientific and medical advancements. However, A2 domain unfolding is made possible after macromolecular unravelling from a globular to a more unraveled conformation occurs, which subsequently exposes A2 domains to hydrodynamic loading. So, before any sort of predictions about the state of constituent A2 domains can be made, the dominant behaviors of the macromolecule occurring in tandem with A2 domain unfolding events must first be characterized. The mechanism responsible for the initiation of macromolecular unraveling in the bulk has been well characterized by a recent protrusion nucleation theory^22–24^, and the characteristics of internal tensile force within sheared multimers has also been studied extensively^8,25,26^. However, the large-scale behaviors and essential macromolecular dynamics of vWF multimers occurring in concert with A2 domain unfolding events is not fully understood.

Characterization of conformational changes on multiple length scales presents numerous hurdles related to the nature of vWF dynamics, as well as their measurement techniques. vWF is a flexible polymer that is capable of undergoing extreme conformational changes on multiple length scales in relatively short periods of time. Traditional methods of extracting the dominant motions from an ensemble of molecular conformations, such as correlation analyses by way of principle component analysis, Cartesian covariance matrices, distance-covariance matrices, or quasi-harmonic analyses, illuminate the “vibrational” motions of the polymer. These methods are often useful for cases where the protein architecture is very structured and the collection of molecular conformations represent samples from short periods of time so that the variance is minimal^27^. One technique extracts dominant conformational motions by means of a correlation analysis using contact matrices, which may be effective in examining the concerted dynamics of the macromolecule and individual A2 domains.

The Random Forest Algorithm (RFA) is a purely data-driven machine-learning tool frequently used to classify and predict system behavior in a wide variety of disiplines^28,29^. A recent RFA application to polymer physics identified that the weakest dihedral angle of a bulk polymer is the most influential molecular feature for determining polymer chain radius of gyration^30^. Another study applied the RFA to account for molecular flexibility of proteins in order to achieve more accurate predictions of protein-protein associations using Monte-Carlo based simulation methods^31^.

In this study, we utilize the RFA to develop a tool capable of predicting the instantaneous A2 domain state based on macromolecular conformational feature information. In doing so, we simultaneously characterize the essential macromolecular dynamics occurring in concert with A2 domain unfolding events by exploiting the nature of the RFA. Further, we identify the most important macromolecular conformational features for the prediction of A2 domain state. The methodological foundations set forth in this work will be extended in a forthcoming study, which will utilize the technique developed here to analyze experimentally obtained data.

## Methodology

### Data acquisition by coarse-grained vWF model simulation

We performed coarse-grained molecular dynamics (MD) simulations using an experimentally parameterized coarse-grain model of vWF in a simple one-dimensional bulk shearing flow, where the flow is in the x-direction and shearing occurs in the z-direction. Data obtained from coarse grained MD simulations were used as input to the RFA. The physical model (see Supplemental Materials) considers a single vWF monomer as a system of two beads connected by a Finitely Extensible Nonlinear Elastic (FENE) spring. The FENE spring represents the A2 domain of the vWF monomer, and has a spring constant and maximum extensible length that were obtained by fitting experimental force-extension data^8,10^. Each bead represents the collection of domains on either side of the A2, and adjacent monomers are connected by stiff harmonic springs that represent disulfide bonds. In this work, we simulated vWF multimers comprised of *N* = 100 beads, which represent proteins 50 monomers in length. Note that this size multimer is in the physiological range of sizes observed for functional vWF in blood, for which the upper limit has been reported to be around 40–200 monomers^1^. 22 non-interacting chains were considered in order to obtain sufficient statistical sampling. Further details and parameters for the molecular model and BD simulations can be found in our previous works^8,21,32^ and in the Supplemental Materials.

### Random forest algorithm

Machine learning encompasses a wide variety of algorithms capable of recognizing structures and patterns in a given dataset. An accurate algorithm for a learning task depends on the biases within the dataset, as well as the type of features to be used as input variables (i.e. numerical or categorical). One major bias is the availability of the true response label, which is the RFA output corresponding to the prescribed input features. This type of bias describes what is called supervised machine learning. The Random Forest Algorithm (RFA) is a tree-based method of supervised learning^33^. The RFA is an aggregation of a large number of decision trees, where each tree is constructed from a bootstrapped sample over the original data. A final decision made by the RFA, or prediction, is based on a majority-wins system among the ensemble of decision trees, where each tree casts one vote. This process introduces a randomness that reduces issues related to overfitting data, and results in increased prediction performance on unseen datasets. As opposed to many other methods, the RFA is capable of providing both an accurate prediction rule and an assessment for the relative importance of predictors^34^. The latter makes this method more appealing for problems related to bioinformatics applications^35,36^.

An essential part of decision tree construction is the splitting criteria that determines features to be used in a hierarchal order. In the current study, the Gini impurity criteria is used and is given by,

$$\sum _{i=1}^{C}{f}_{i}(1-{f}_{i})$$

where *C* is the number of unique labels and *f*_*i*_ is the frequency of label *i* at a node. This criterion also enables the importance of each feature to be ranked based on decreased node impurity. We implemented the RFA from the Scikit-learn module^37^.

### Features and responses

In this study, we trained the RFA with a set of input variables obtained from data generated by coarse-grained MD simulations of vWF in shear flows. These input variables are macromolecular conformational features listed in Table 1. The training data set was comprised of 200,000 uncorrelated feature observations. One-half of the data corresponded to polymeric conformations for which no A2 domains were unfolded, and the remaining half corresponded to chains for which at least one A2 domain was unfolded. 70% of the total data were used for RFA training, and 30% were used for prediction testing as unseen data. Three sets of input features/responses were used for separate trainings that depended upon resolution of the feature information: chain-level, 5-segment, and 10-segment partition resolutions were used. The chain-level resolution considered the entire chain as a single partition, so that features characterized conformational information for the entire multimer. Additionally, the multimer was partitioned along its contour into 5 and 10 segments of equal length (i.e. 20 and 10 beads per partition for the 5- and 10-segment resolutions, respectively). In these cases, features for all segments were used in RFA training, but input variables characterized macromolecular conformational information for a single partition only. In addition, the full set of Cartesian bead positions for the ensemble were also included as input features for all three resolution levels. However, ensemble bead positions were excluded from the partitioning process, so that each individual bead position component was included as an input feature to the RFA. Note that we have succinctly expressed the ensemble bead position features by three indices that represent the sum of the x-, y-, and z-components of all bead positions.

**Table 1:** Input features used for random forest algorithm training.

Feature Information				
Feature Name	Symbol	Feature Indices		
		Chain-Level	5-Segment	10-Segment
Sum of x-Direction Bead Positions	Σ*r*_*X*_	1		
Sum of y-Direction Bead Positions	Σ*r*_*Y*_	2		
Sum of z-Direction Bead Positions	Σ*r*_*Z*_	3		
x-Direction Velocity	*v*_*X*_	4	4–8	4–13
y-Direction Velocity	*v*_*Y*_	5	9–13	14–23
z-Direction Velocity	*v*_*Z*_	6	14–18	24–33
Flow-Induced Bead Displacement	Δ*r*	7	19–23	34–43
Maximum x-Direction Distance	*MAX*_*X*_	8	24–28	44–53
Maximum y-Direction Distance	*MAX*_*Y*_	9	29–33	54–63
Maximum z-Direction Distance	*MAX*_*Z*_	10	34–38	64–73
Multimer End-to-End Distance	*EE*	11	39–43	74–83
x-Direction Radius of Gyration	*RG*_*X*_	12	44–48	84–93
y-Direction Radius of Gyration	*RG*_*Y*_	13	49–53	94–103
z-Direction Radius of Gyration	*RG*_*Z*_	14	54–58	104–113
Spherical Radius of Gyration	*RG*_*TOT*_	15	59–63	114–123
Multimer Variation Fraction in Flow-Direction	*VFF*	16	64–68	124–133
Local Chain Concentration	*LCC*	17	69–73	134–143
First Principle Component Projection in Flow-Direction	*PCA*_1_	18	74–78	144–153
Second Principle Component Projection in Flow-Direction	*PCA*_2_	19	79–83	154–163
Third Principle Component Projection in Flow-Direction	*PCA*_3_	20	84–88	164–173

The instantaneous A2 domain state was used as the response for training, which was either 1 or 0 for an unfolded or folded A2 domain, respectively. A single response was given for the chain-level resolution, whereas 5 and 10 binary responses were used for the 5- and 10-segment resolution levels, respectively. The physical model employed two beads and one FENE spring to describe the vWF monomer, so a partition within the 5-segment resolution comprised of 20 beads had 10 connecting FENE springs capable of unfolding. If any of the A2 domain represented FENE springs within a given partition were found to be unfolded, then the response assigned to this partition was 1. If instead all constituent A2 domains were folded at an instant, then the response assigned to that partition was 0. Results obtained from single-molecule pulling experiments found that the most likely unfolding force for the A2 domain is approximately 11 *pN*^10^, although A2 domain unfolding events in experiment were observed over a range of force values between 7–15 *pN*. Accordingly, we define A2 domain unfolding to be any force excursion event of FENE spring tension past the experimentally-motivated value of 11 *pN*, which is in the middle of the range reported by experiment.

We were motivated to examine three partition resolutions for a few reasons. Firstly, we suspected that prediction performance of the RFA could be increased if feature/response information was more finely resolved along the multimer contour, which encouraged us to partition the chain into many segments. However, we desired to develop a procedure relevant to both simulation and experiment, which limited the number of possible partitions to avoid being devoid of experimental applicability. Various regions of a polymer exhibit different fundamental behavior that is dependent upon their location along the chain contour, which we knew a priori. Accordingly, we partitioned the multimer into numerous segments in an attempt to extract these position-dependent dynamics from within the data using the RFA. Further, we desired to identify possible correlations in macromolecular behavior among these segments, which necessitated the RFA to distinguish features and responses from the various segments.

The notion of position-dependent dynamics is well-illustrated by two examples: protrusion nucleation and hydrodynamic shielding. Macromolecular unraveling is initiated when a small thermally-nucleated protrusion emerges from an otherwise globular multimer^22–24^. The protrusion is subsequently exposed to hydrodynamic force, which may lead to complete macromolecular unraveling if the protrusion continues to elongate due to a sufficiently strong local flow field. In the case of protrusion nucleation, the behavior at the multimer termini is what causes macromolecular unraveling, whereas the dynamical behavior near the multimer center is what follows in effect. Another location dependent behavior is exemplified by shielding effects induced by hydrodynamic interactions among members of the chain. Shielding effects describe a situation where one bead shields another so that the presence of one bead mitigates the hydrodynamic force felt by another. These hydrodynamic interactions are actually disturbance velocities propagated through the flow field due to the forced motion of beads^38,39^. The effect of such disturbances depends on the separation distance between the hydrodynamically interacting beads, and can manifest different behaviors along the mutlimer contour as a result.

Conformational feature information listed in Table 1 is described below in more detail. Each individual bead position component was given as input to the RFA. However, we succinctly refer to these 300 features (3 spatial coordinates for each of the 100 beads comprising the multimer) by the sum of the x-, y-, and z-direction position components, given by indices 1–3. For the remaining features listed in Table 1, one value was given as input to the RFA for each segment of the chain, which depends upon the resolution level (i.e. 5 and 10 values per feature for the 5- and 10-segment partition resolutions). Velocity component features describe the center-of-mass velocity for a given partition. The flow-induced bead displacement is the center-of-mass displacement of a given partition purely due to the undisturbed velocity field acting on beads. Maximum distance component features are defined by the maximum distance between any, but not necessarily the same, two beads in the x-, y-, and z-directions for a given partition. The end-to-end distance is the distance between the first and last bead of a given partition. Radii of gyration components define the spatial variance of bead positions for a given partition in the standard Cartesian system, or equivalently they measure the length of three radii that characterize the segment conformation using an ellipse oriented along the x-, y-, and z-axes. The spherical radius of gyration is similar, but measures the radius of a sphere that describes partition conformation. The multimer variation fraction in the flow-direction measures the fraction of spatial variation exhibited by the partition that is in the flow direction. It is defined by the flow-direction radius of gyration (that is the x-direction radius of gyration for the simple flow field herein considered) divided by the sum of the radii of gyration in the x-, y-, and z-directions – it gives a measure of flow-induced variation relative to the total variation of spatial distribution for a given partition. The local chain concentration, or density, gives a measure of local polymeric packing. A threshold distance measured from the partition center-of-mass was prescribed, which was 50, 10, and 5 bead radii for the chain-level, 5-segment, and 10-segment partition resolutions, respectively. The ratio of threshold distance, measured in bead radii, to number of beads per partition is 1/2 for all resolution levels. The local chain concentration is defined by the number of beads from any segment existing within this threshold distance from the partition center-of-mass, normalized by the total number of beads in the ensemble. Lastly, a principle component analysis was performed among the beads comprising a given partition, and three principle component vectors were obtained. These vectors describe three orthogonal directions in Cartesian space that most completely capture the variation in the spatial distribution of partition members. In other words, they point in the directions of space that the backbone of the polymer also points toward, and they are ordered in descending importance (i.e. the first principle component vector is the most informative). Accordingly, the last three features listed in Table 1 indicate the projections of the three principle component vectors in the flow-direction, which is actually equal to the cosine of the angle between them since the PCA vectors and the flow-direction vector have unit magnitude.

## Results and Discussion

### Prediction performance

**Figure 1:**
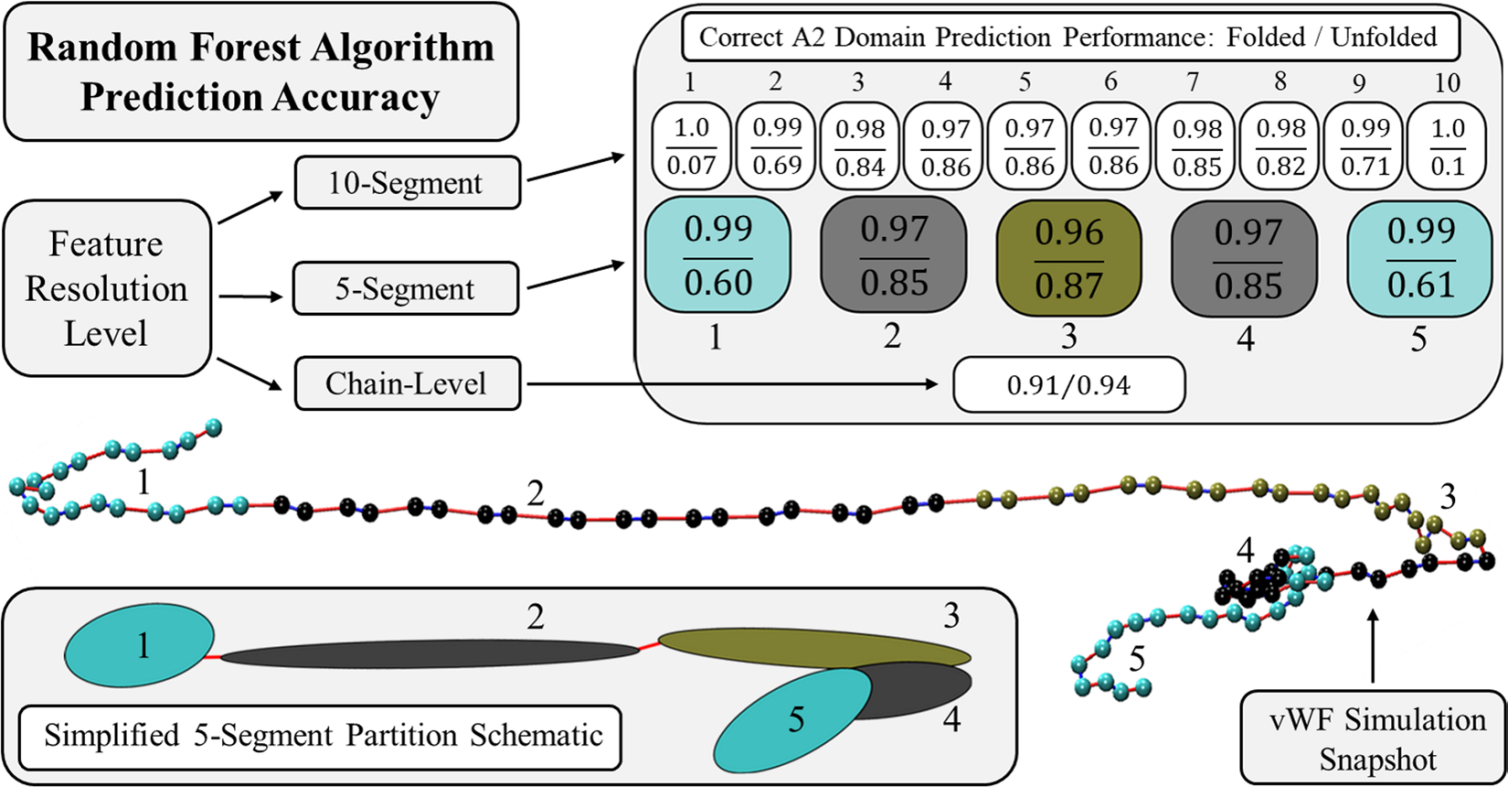
Random Forest algorithm prediction performance is tabulated in the upper right sub-table for three feature/response partition resolutions considered: 10-segment, 5-segment, and chain-level. Column entries correspond to a single segment along the chain contour wherein features/responses are calculated. Performance ranges between 0 and 1, where 1 indicates perfect predictive performance. Two performance values are reported for each partition indicating correct prediction of (top) folded and (bottom) unfolded A2 domains. Below is a simulation snapshot of an unraveled vWF multimer in a shearing flow, where bead colors are used to delineate partition members. An oversimplification of chain partitioning is shown by the cartoon schematic in the lower left corner. Colors and numeric labels in the table, snapshot, and schematic are used to differentiate segments along the multimer contour for the 5-segment case, and are consistent throughout this study for all reported results and figures.

Figure 1 tabulates the RFA prediction performance for determining the state of constituent A2 domains for three feature/response resolution levels. Performance ranges between 0 and 1, where 1 indicates perfect predictive performance. Two values are reported for algorithm performance that indicate correct prediction of (top) folded and (bottom) unfolded A2 domains. Also illustrated in Fig. 1 is a simulation snapshot of vWF in a bulk shearing flow^40^.

Correct prediction of folded A2 domains for the 5- and 10-segment resolutions is highly accurate at the multimer termini (∼98–100%), and diminishes slightly nearer to the chain center (∼96–97%). The opposite trend is observed for correct prediction of unfolded A2 domains. Partitioning with 10-segments yielded 7% and 10% accuracy in unfolded A2 domain predictions for the outermost segments. This dramatically increased for their neighbors to 69% and 71%, and was maximal near the multimer center at 86% accuracy. Prediction accuracy at the multimer center is nearly identical for both the 5- and 10-segment resolutions (∼86–87%). However, unfolded A2 domain predictions in the outermost partitions for the 5-segment case exhibit tremendous improvement compared to the 10-segment case with 60% and 61% prediction accuracy. Prediction performance at the chain-level resolution is high for folded and unfolded A2 domain states at 91% and 94% accuracy, respectively. The accuracy of predictions at the chain-level resolution are considerably high, but at a cost to knowledge of A2 domain state location along the multimer contour. However, prior authors have advanced that unfolding of the A2 domain is the rate-limiting mechanism in vWF cleavage by the enzyme ADAMTS13^16,25^, and therefore any A2 unfolding event can be associated with subsequent cleavage of the vWF multimer. In this case, a practitioner may only be concerned if A2 domain unfolding occurs whatsoever, and the location of the unfolded A2 domain(s) may be of less importance.

Figure 1 illustrates the high accuracy of the RFA for predicting A2 domain state based on the macromolecular conformational feature information considered here. Proof-of-concept has been done using simulation-generated data, and is promising for the analysis of experimental data. Clearly not all of the features considered in this work have experimental relevance however. Nonetheless, some of the existing features can be extracted from single-molecule flow experiments using fluorescence microscopy imaging, such as the local chain concentration, in-plane position, 2 of 3 principle component projections, end-to-end distance, and the x- and y-direction maximum separation distances. However, it may not be possible to calculate these feature values to arbitrary resolution (i.e. number of partitions), especially given that the theoretical limit of fluorescence microscopy imaging equipment is of order 200 nm and molecular diffusion can cause blurring during the image acquisition time. Fu *et al*. analyzed fluorescence distributions along unraveled vWF multimer contours, and estimated the average extension per monomer with a resolution of tens of nanometers^41^. Molecular tension sensors with single piconewton sensitivity have been developed for use in living cells by fluorescence force spectroscopy^42,43^. Scanning angle interface microscopy permits for the measurement of molecular height in the direction normal to the scanning plane, and can determine the inclination of unraveled surface-bound polymer chains relative to the shearing lamina with nanometer resolution^44^. Finally, A2 domain state can be probed using a fluorescent molecular tag capable of specifically binding to the scissile bond site within the A2 domain structure. Theoretically, a series of microfluidic flow experiments using fluorescence microscopy imaging techniques could be devised that estimate A2 domain state and the values of predetermined features characterizing flow-induced conformational changes and internal stresses, which occur on length scales as little as tens of nanometers and as large as ten microns. This feature/response data could be analyzed using a RFA to better understand the configurational landscape of the macromolecule associated with sub-monomer A2 domain unfolding events, and in theory, make predictions about such multi-scale behaviors.

The low prediction accuracy for unfolded A2 domains in the outermost partitions, shown in Fig. 1, bears further discussion. Non-grafted linear polymer chains immersed in a homogenous flow are subject to external frictional forces that must be internally balanced. Under the influence of strong shearing flow, flexible polymer chains freely tumble through the fluid medium while continuously transitioning between globular and elongated conformations. Terminal segments of the polymer have fewer topological constraints because of their one-sided connectivity, and can more readily dissipate frictional forces through spatial translation or realignment with the flow direction – as opposed to FENE spring extension.

This is evidenced by the ensemble average FENE spring tension distribution along the multimer contour (not illustrated, see our prior work^8^), which is parabolic with little force at the termini and peak force at the center. In this work, we have defined A2 domain unfolding to be any force excursion event of FENE spring tension past the experimentally-motivated value of 11 *pN*. While this definition is certainly a gross simplification of what is actually a sudden, probabilistic, and dramatic unfolding event, the FENE spring model reliably captures smoothed force-extension correlations of unfolded A2 domain dynamics^8,21^. In light of the parabolic tension distribution and definition for unfolding, it is intuitive that the probability for A2 domain unfolding (i.e. significant force excursion events) also exhibits a quasi-parabolic distribution along the chain contour (not illustrated, see our prior work^8^). Hydrodynamic forces induce little internal tension near the polymer termini so that the probability of unfolding is small there, however force magnitude and the likelihood of unfolding increase towards the multimer center. Among the 140,00 observations used for training in the 10-segment case, only 0.4% of the data represented conformations with unfolded A2 domains in the terminal segments, whereas this value was over 20% for the two central segments. The 50-fold difference in A2 domain unfolding statistics between terminal and central segments justifies the low prediction accuracy for unfolded A2 domain states at the 5- and 10-segment resolution levels, as compared to the chain-level resolution.

We simulated vWF multimers at the large shear rate of $\dot{\gamma }=500\times {10}^{3}{s}^{-1}$ in order to ensure a high frequency of A2 domain unfolding events for RFA training. We intentionally introduced a statistical bias when defining the training data set by selecting one-half of the input data to correspond to conformations for which no A2 domains were unfolded, and the other half corresponding to conformations for which at least one A2 domain was unfolded. We expect that prediction accuracy near the multimer termini for unfolded A2 domain states can be improved dramatically by introducing an additional statistical bias to the training data set, and increasing its size. For example, we could have selected an ensemble of observations that, cumulatively, represented an equal number of unfolded A2 domains in each segment, rather than by randomly sampling conformations with at least one A2 domain unfolded. However, the aim of the current study is not to optimize an algorithm, but rather to demonstrate the use of a RFA for predicting the dynamics of linear polymers with sub-monomer features based on macromolecular conformational feature information.

Additionally, we do not wish to have in depth discussion of the biological or physiological implications of our coarse-grained BD simulation results, but rather on the development and findings of a RFA that uses such results as input. Nonetheless, we will state that there is no direct experimental evidence showing that A2 domains located nearer to the multimer termini of non-grafted vWF molecules in bulk shearing flows are more likely to be in a folded state. However, provided that the tension distribution in the regime of large shearing is parabolic, this is what is suggested by our BD simulation results and was the claim made in one of our previous works^8^. The assertion that terminal A2 domains are more probably in a folded state is reasonable in light of considering a more robust description of A2 domain unfolding – as an activated process, whereby transition of an A2 domain from the folded to unfolded state is separated by a transition state energy barrier that is lowered by the application of an external force.

### Cumulative feature importance

**Figure 2:**
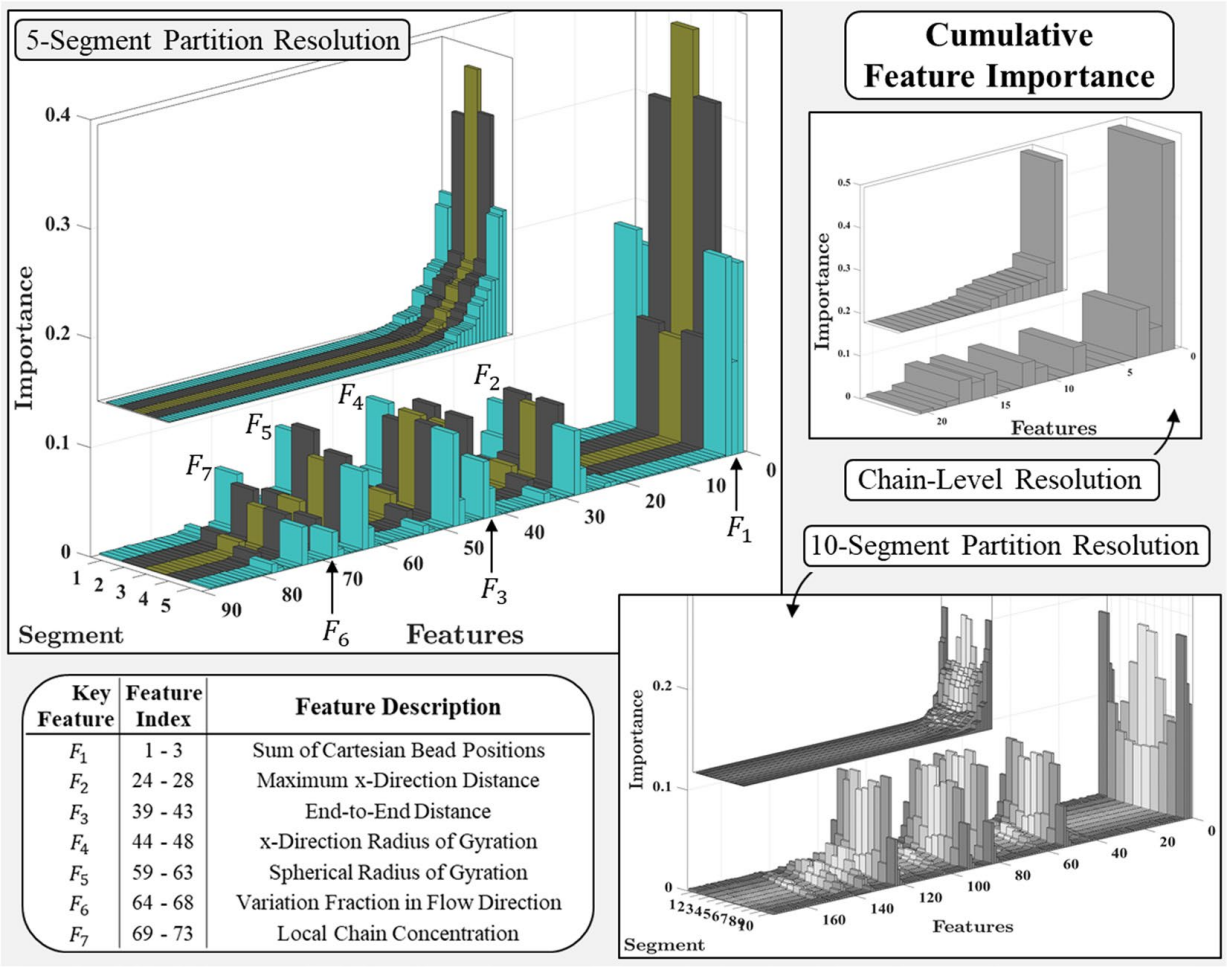
Feature importance scores for Random Forest Algorithm input variables. Results are illustrated for three feature/response partition resolutions considered: 10-segment, 5-segment, and chain-level. Feature indices for bottom bar graphs correspond to feature names listed in Table 1, and top bar graphs illustrate the same data, but sorted in order of ascending importance. Features showing notable importance are identified and tabulated for the 5-segment resolution case.

Figure 2 illustrates feature importance scores determined by the RFA for prediction of A2 domain states. Results for three feature/response resolutions are shown in Fig. 2, and the bottom bar graphs for each resolution have feature indices that correspond to feature names given in Table 1. The top bar graphs for each resolution level illustrate the same data, but sorted in order of ascending importance to illustrate that a relatively small fraction of all features considered account for a majority of the total prediction importance. Features of notable importance for the 5-segment resolution level are also tabulated in Fig. 2. Note that several of these key features may exhibit collinearity, but we have not examined this possibility or its effects because that is outside the scope of this work and will be done in a future study. The simple case considered in this work examined a vWF multimer subject to a bulk shearing flow in the x-direction, however the selected features may not be collinear when simulating more complex flow conditions such as vWF multimers in the presence of a wall, ancillary particles, or more complex flow fields.

Table 2 lists the 14 most important macromolecular conformational features for prediction of A2 domain state within each partition of the 5-segment resolution. They are easily seen in the top bar graph of the 5-segment partition results in Fig. 2. The total importance accounted for by the 14 most important features is listed in Table 2 for each segment. The total number of features considered for the 5-segment resolution is 88, and Table 2 shows that a relatively small subset of them (14 out of 88) account for 87–94% of the total prediction importance, depending on segment location. The prediction importance accounted for by the 14 most important features is lower for segments nearer to the multimer termini. Accordingly, the prediction importance is less isolated to the most significant features, and distributed more among the others. This is caused by what we refer to as correlated segment dynamics. This means that prediction of A2 domain state in one segment depends not only upon the feature characteristics of that segment, but also upon features that characterize neighboring segments. Cross-correlations have little influence on A2 domain state prediction at the multimer center, indicated by the 14 most important features for segment 3 accounting for 94% of the total importance and being solely related to feature information of segment 3 (except for Σ*r*_*x*_, Σ*r*_*y*_, and Σ*r*_*z*_ that transcend segment classification). Alternatively, 4 out of the 14 most important features for the first and last segments characterize information pertaining to neighboring segments. Correlated segment dynamics and their implications will be discussed further throughout the remainder of this work.

**Table 2:** Fourteen most important macromolecular conformational features for prediction of A2 domain state in each partition of the 5-segment resolution, as well as their cumulative importance.

Feature Importance Rating	Segment				
	1	2	3	4	5
14	*Rg*_Y,1_	***MAX***_***X*,3**_	*Rg*_Z,3_	***EE***_**3**_	*MAX*_*Y*,5_
13	***Rg***_**X,2**_	***EE***_**3**_	*MAX*_*Z*,3_	*PCA*_1,4_	***Rg***_**,TOT,4**_
12	***Rg***_**TOT,2**_	*PCA*_1,2_	*PCA*_1,3_	***MAX***_***X*,3**_	***Rg***_**X,4**_
11	*VFF*_1_	*MAX*_*Y*,2_	*MAX*_*Y*,3_	*MAX*_*Y*,4_	***MAX***_***X*,4**_
10	***MAX***_***X*,2**_	*Rg*_Y,2_	*Rg*_Y,3_	*Rg*_Y,4_	*VFF*_5_
9	***EE***_**2**_	*VFF*_2_	*LCC*_3_	*VFF*_4_	***EE***_**4**_
8	*EE*_1_	*LCC*_2_	*VFF*_3_	*LCC*_4_	*LCC*_5_
7	*MAX*_*X,1*_	Σ*r*_*Y*_	Σ*r*_*Y*_	Σ*r*_*Y*_	*EE*_5_
6	*LCC*_1_	*MAX*_*X*,2_	*Rg*_TOT,3_	*MAX*_*X,4*_	*MAX*_*X,5*_
5	*Rg*_TOT,1_	*Rg*_X,2_	*Rg*_*X*,3_	*Rg*_TOT,4_	*Rg*_TOT,5_
4	*Rg*_X,1_	*EE*_2_	*EE*_3_	*Rg*_X,4_	Σ*r*_*Y*_
3	Σ*r*_*Y*_	*Rg*_TOT,2_	*Rg*_X,3_	*EE*_4_	*Rg*_X,5_
2	Σ*r*_*X*_	Σ*r*_*Z*_	Σ*r*_*Z*_	Σ*r*_*Z*_	Σ*r*_*X*_
1	Σ*r*_*Z*_	Σ*r*_*X*_	Σ*r*_*X*_	Σ*r*_*X*_	Σ*r*_*Z*_
	0.87	0.91	0.94	0.91	0.87
	**Prediction Importance of 14 Most Important Features**				

Features in bold font and highlighted cells exemplify correlated segment dynamics, which manifest simultaneously with sub-monomer A2 domain unfolding events in a given partition along the contour. Numerical subscripts indicate the segment location of variable measurements.

### Position importance distribution along multimer contour

**Figure 3:**
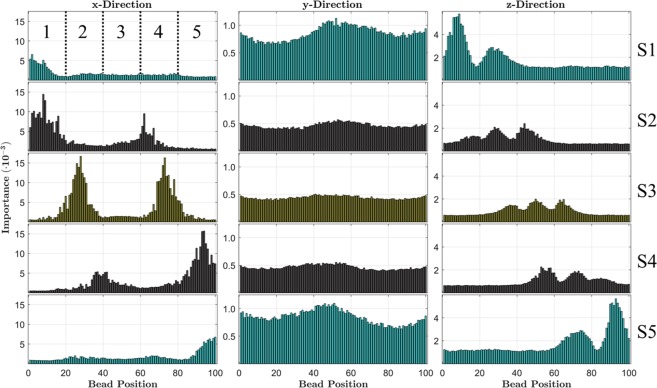
Distribution of x-, y-, and z-direction bead position importance scores along the multimer contour for the 5-segment partition resolution. The sum of these distributions are indexed by labels 1, 2, and 3 in Table 1, respectively. Rows, labeled S1-S5, indicate prediction importance distributions for each segment (i.e. the x-direction distribution within row S1 illustrates the importance distribution of only x-direction bead positions for determining A2 domain unfolding within segment 1 only).

Figure 3 illustrates bead position importance scores along the vWF multimer contour. Columns in Fig. 3 illustrate prediction importance for the x-, y-, and z-direction position components for each segment, where segments are indicated by rows labeled S1–S5. Our previous results shown in Fig. 2 and Table 2 have dealt with *cumulative* feature importance, which treated the sums of ensemble position components as single features for each segment. However, Fig. 3 illustrates the distribution of importance along the multimer contour, which clearly shows prevalent correlated segment dynamics. For clarity, the jargon used to interpret results illustrated in Fig. 3 for the last row of the last column, as an example, is that this distribution illustrates the prediction importance scores for all z-direction bead position component features for determining A2 domain unfolding only within segment 5 of the vWF multimer.

Our motivation for the explicit inclusion of each position component is made evident by the results illustrated in Fig. 3. We have exploited our knowledge of the instantaneous A2 domain states to utilize the RFA as a tool for building a statistical description of the essential macromolecular dynamics occurring in tandem with A2 domain unfolding events. Locations of high positional importance are, in some way, correlated with A2 domain unfolding events and importantly show strong evidence of cross-correlations in macromolecular behavior. The results in Fig. 3 indicate *where* to look for important macromolecular conformational information, which reduced the task to merely determining *what* that information is. Our domain knowledge of the underlying polymer physics combined with a backwards working approach revealed the nature of the underlying large scale motions that facilitate A2 domain unfolding as a function along the chain.

Based on analysis of feature values and visualization of polymer conformations, we found that the inhomogeneous distributions of positional importance are easily corroborated by elementary polymer physics principles. Two types of behavior are evident in the x-direction position importance distributions in the first column of Fig. 3. Unfolding within the outermost partitions occurs most often at the interior region farthest from the mutlimer terminus, because internal tension within the outer segments becomes highest there. Accordingly, beads located nearer to the multimer termini exhibit large x-direction position importance, because the location of these beads relative to the rest of the chain determines the overall segment elongation in the flow direction, shown in rows S1 and S5 of the first column. A2 domain unfolding is strongly correlated with large segment elongations in the flow direction, because A2 domains are exposed to the force of flow after segment unraveling has occurred. A2 domain unfolding within an interior partition requires the two adjacent segments to “pull” on it in the flow direction from either side, which explains the double-peak structures shown in rows S2–S4 of the first column. Two trends are exhibited in the z-direction position distributions in the third column of Fig. 3. The outermost partitions show two peaks in z-direction position importance, whereas interior partitions show three peaks. In both cases, the relevant physical principle is that two or more regions of the multimer must become skewed to the flow in the z-direction (shearing-direction) in order for sufficient hydrodynamic force to be exerted upon constituent A2 domains. Adjacent regions of the chain with differing z-direction heights exist within different shear lamina of the flow, which causes a net hydrodynamic force to act on these regions that drives multimer elongation and local A2 domain unfolding. Feature importance for y-direction position components, shown in the second column of Fig. 3, are collectively more important for the outermost partitions than interior. The strength of shearing is proportional to relative displacements in the z-direction, so elevation differences along the multimer can induce dramatic changes in polymer conformation. Since terminal segments of the multimer are subject to fewer topological constraints, members of the outermost partitions are more able to diffuse in the xy-plane to maintain lower energy configurations by minimizing the span of shear lamina. Interior partitions are physically connected at both ends, which restricts this sort of planar diffusion and explains the elevated importance for y-direction position components in the terminal segments. Two figures similar to Fig. 3 are included in the Supplemental Material (Figs S2, S3) for the 10-segment and chain-level partition resolution. Fig. S2 illustrates the same story conveyed here, however the z-direction position component distributions for terminal segments exhibit a single peak and interior segments exhibit two peaks.

Correlated segment dynamics are readily observable in the distributions of Fig. 3. For example, the x-direction distribution for segment 3 (row S3, first column) illustrates that the importance of bead positions within this segment is more than 11 times *less* important than the position of beads in neighboring segments 2 and 4. An A2 domain was considered to be unfolded if the internal tensile force within the connecting FENE spring was greater than 11 *pN*, and the FENE force value was determined solely by the bead-bead separation distance that defines the FENE spring extension. Therefore, one may falsely assume that our choice to explicitly include each bead position component as an input feature to the RFA would ensure highly accurate predictions merely because the response is a sort of non-linear transformation of the features. However, the x-direction distribution for segment 3 shows that this is certainly not true; rather, the position of distant beads that are not physically connected to the unfolding domain are dramatically more important for predicting A2 domain dynamics. As we have shown in Fig. 1, the RFA is capable of making highly accurate predictions, but not because of first-order correlations among the features and response. Instead, accurate predictions are possible, in part, because higher-order correlations within the data due to the concerted macromolecular dynamics occurring among distant segments of the chain are readily recognized and understood by the RFA. This result highlights the critical importance and prevalence of correlated segment dynamics in vWF multimers subject to bulk shearing flows, as well as the usefulness of machine learning techniques to elucidate these seemingly hidden behaviors.

### Further examination of correlated segment dynamics

**Figure 4:**
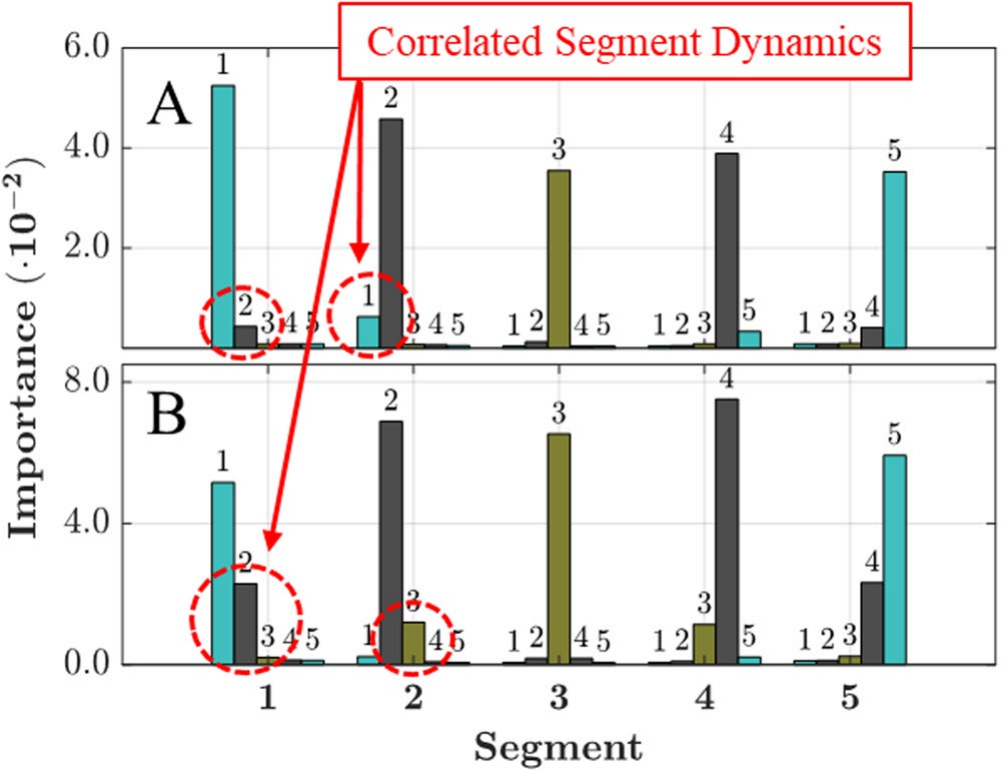
Feature importance scores for (**A**) local chain concentration and (**B**) maximum x-direction distance within each partition of the 5-segment resolution. Feature cross-correlations are highlighted by dashed circles.

Figure 4 illustrates two features exhibiting correlated segment dynamics: (A) local chain concentration and (B) maximum x-direction distance for the 5-segment resolution level. Nearly all of the key features identified in the sub-table of Fig. 2 exhibit some degree of cross-correlation, but with varying extent. Cross-correlations among bead position components were previously discussed and illustrated in Fig. 3. The general trend observed for the remaining features is that prediction importance depends on the value of a given feature within that partition, but also on the value of that same feature in the adjacent segment that is nearer to the multimer center. The value of the same feature in the adjacent segment that is nearer to the multimer termini appears to be negligibly important. However, the local chain concentration feature deviates from this general trend and is illustrated in Fig. 4A. Although the magnitude of the dependencies is small, the local chain concentration importance, shown in Fig. 2A, for segments 2 and 4 clearly show cross-correlations with segments segment 1 and 5, respectively, which is in the opposite direction suggested by the general trend.

## Conclusions

We have shown that the instantaneous state of sub-monomer A2 domains can be accurately predicted based on features that characterize macromolecular conformation information of von Willebrand Factor multimers in shear flows. Our method employed a purely data-driven machine learning technique, whereby the Random Forest Algorithm was trained using three data sets of feature/response information of varying resolution detail. Post-training predictions were made using an unseen data set. The accuracy of these predictions for unfolded A2 domains ranged from 86–94% at the multimer center, depending on the feature/response resolution level considered, with a certainty of their location resolved to as little as 5 out of 50 possible A2 domains along the vWF multimer contour. The high accuracy of predictions made indicates that the feature information used as input variables to the RFA sufficiently describes the behavior of vWF in shearing flows. We identified the most important features for prediction of A2 domain state, and found that a small fraction of the total features considered account for the majority of the prediction importance – the 14 most important features out of 88 total features for the 5-segment resolution level accounts for 87–94% of the total prediction importance, depending on segment location. By exploiting the nature of the Random Forest Algorithm, we extracted the dominant motions of the vWF multimer occurring in tandem with A2 domain unfolding events from an ensemble of macromolecular conformations. We observed that these essential large-scale molecular motions manifest in strongly correlated dynamics among various segments of the vWF chain, which occur in concert to facilitate A2 domain unfolding. We found that neighboring regions of the multimer adjacent to an A2 domain unfolding event are required to assume elevation differences in the shearing direction, which causes a local hydrodynamic loading that induces elongation in the flow-direction. Accordingly, we found that A2 domain unfolding requires neighboring regions of the chain on either side to “pull” on it in the flow-direction. Aside from the strong correlations observed among bead position features (or equivalently the chain *configuration*), we showed that correlations among individual segment dynamics have stronger influence in the outermost partitions as compared to centrally-located regions. Here, a procedure has been advanced that permits significant dimensionality reduction from an entire coarse-grain simulation ensemble to a small collection of key observables at the macromolecular scale, which permit for high accuracy prediction of sub-monomer response. To further demonstrate the impact of such dimensionality reduction, in a forthcoming work, the procedure developed here will be implemented to analyze macromolecular conformation information from experimentally generated data^45^.

## Supplementary information

Supplemental Materials

## Data Availability

Data generated for use in the current study, and data generated by the procedure herein conducted, are available from the corresponding author on reasonable request.
