# Supplementary material for: Prediction of Sub-Monomer A2 Domain Dynamics of the von Willebrand Factor by Machine Learning Algorithm and Coarse-Grained Molecular Dynamics Simulation
Source: Sci Rep. 2019 Jun 21;9:9037. doi: 10.1038/s41598-019-44044-2 (PMC6588549; doi:10.1038/s41598-019-44044-2)
Supplement: Supplementary file 1 — Supplemental Materials [file 41598_2019_44044_MOESM1_ESM.pdf]

Supplemental Materials

for

Prediction of Sub-Monomer A2 Domain Dynamics of the von Willebrand Factor by Machine Learning Algorithm and Coarse-Grained Molecular Dynamics Simulation

by

Michael J. Morabito, Mustafa Usta,
Xuanhong Cheng, Xiaohui F. Zhang, Alparslan Oztekin, and Edmund B. Webb III

Coarse-Grained vWF Monomer Model

Figure S1 depicts the domain-level molecular modeling process, which results in a parameterized bead-spring model of the vWF monomer.

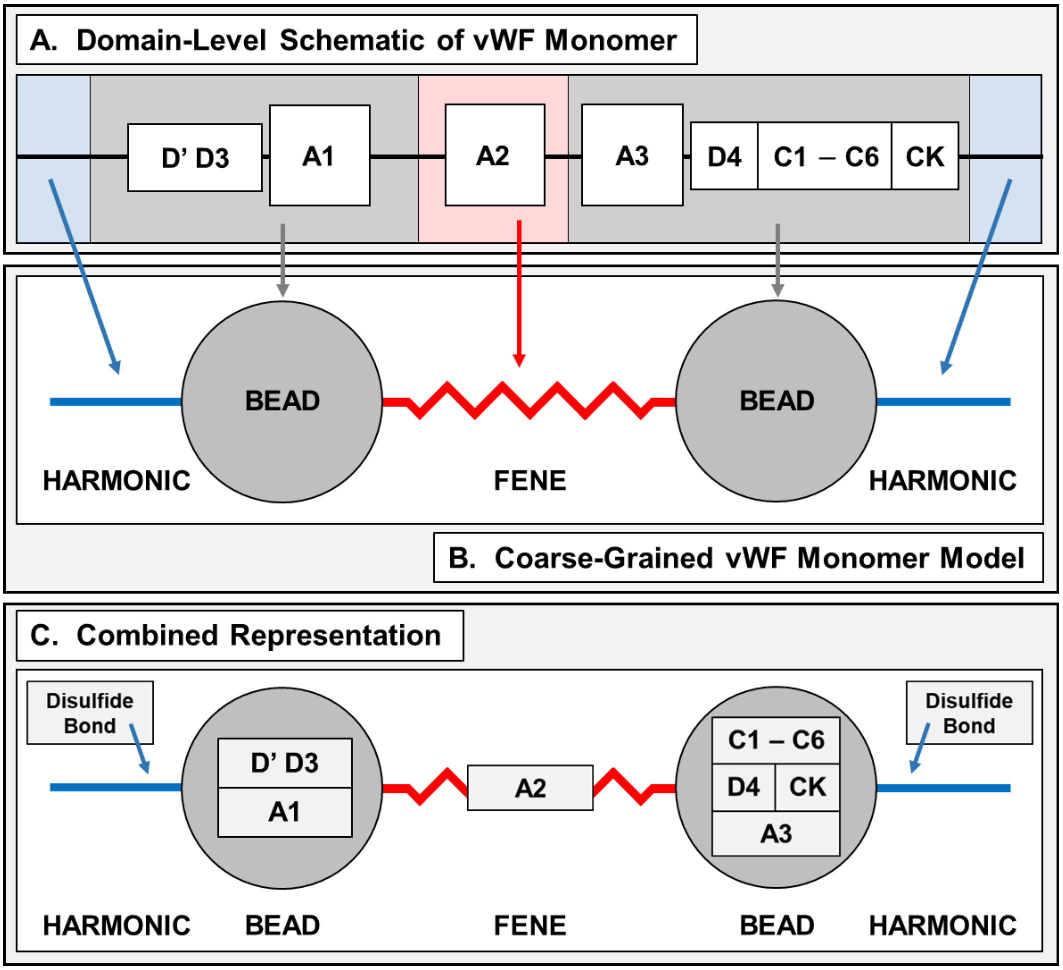

Figure S1. (A) Schematic of the domains or domain assemblies comprising one vWF monomer. The background coloring corresponds to the monomer model coloring used in (B – C). (B) Bead-spring model employed to represent the vWF monomer. (C) Each spherical bead (gray) has a radius of $a = 15\,nm$, and represents the groupings of domains on either side of the A2 domain. The A2 domain is explicitly represented by a finitely extensible nonlinear elastic (FENE) spring (red). Adjacent monomers are connected by stiff harmonic springs (blue), which represent strong disulfide bonds.

Brownian Dynamics Simulations

Langevin Equation

Results from coarse-grained molecular dynamics simulations were used as input variables to the random forest algorithm. Brownian dynamics simulations for the bead-spring model are performed using the discretized Langevin equation,

$$\vec{\boldsymbol{r}}_i^{new} = \vec{\boldsymbol{r}}_i^{old} + \left[(\boldsymbol{\nabla}\vec{\boldsymbol{u}}^{\infty})^T \cdot \vec{\boldsymbol{r}}_i + \frac{1}{k_b T}\sum_{j=1}^{N} \underline{\boldsymbol{D}}_{ij} \cdot \left(\vec{\boldsymbol{F}}_j^{LJ} + \vec{\boldsymbol{F}}_j^{S}\right)\right]\Delta t + \vec{\xi}_i \ .$$

The position vector of the ith bead, $\vec{\boldsymbol{r}}_i^{new}$, is given by the position at the previous time step, $\vec{\boldsymbol{r}}_i^{old}$, plus a bead displacement occurring over the time step, Δt. $k_b = 1.381 \cdot 10^{-2}\frac{pN\,nm}{K}$ is Boltzmann's constant and $T = 300\ K$ is the temperature.

$(\boldsymbol{\nabla}\vec{\boldsymbol{u}}^{\infty})^T \cdot \vec{\boldsymbol{r}}_i\,\Delta t$ represents the displacement due to the unperturbed solvent velocity, $\vec{\boldsymbol{u}}^{\infty}$, at the position of the ith bead. $(\boldsymbol{\nabla}\vec{\boldsymbol{u}}^{\infty})^T \cdot \vec{\boldsymbol{r}}_i = \dot{\gamma} z \hat{\boldsymbol{x}}$ for the simple shearing flows herein considered, where $\dot{\gamma}$ is the shear rate, z is the height of the ith bead in the shearing direction along the positive z-axis, and $\hat{\boldsymbol{x}}$ is a unit vector pointing in the direction of flow along the positive x-axis.

$\frac{1}{k_b T}\sum_{j=1}^{N} \underline{\boldsymbol{D}}_{ij} \cdot \left(\vec{\boldsymbol{F}}_j^{LJ} + \vec{\boldsymbol{F}}_j^{S}\right)\Delta t$ represents the displacements due to systematic forces acting on the ith bead plus the $N-1$ disturbance velocities that are generated by the forced motion of neighboring beads, propagated through the solvent, and realized at the position of $\vec{\boldsymbol{r}}_i$. $\underline{\boldsymbol{D}}_{ij}$ is the second-order diffusion tensor that accounts for Hydrodynamic Interactions (HI), and is multiplied by the systemic bead forces that are defined below.

$\vec{\xi}_i$ is a stochastic displacement that accounts for hydrodynamic interactions among the interacting Brownian particles. The random Brownian bead displacement is given by,

$$\vec{\xi}_i = \sqrt{6\Delta t}\sum_{j=1}^{i} \underline{\boldsymbol{\sigma}}_{ij} \cdot \vec{\boldsymbol{n}}_j \ .$$

$\underline{\boldsymbol{\sigma}}$ is the $3N \times 3N$ Brownian tensor obtained by taking the square-root of the fourth-order diffusion tensor, $\underline{\boldsymbol{D}}$, by Cholesky decomposition. $\underline{\boldsymbol{\sigma}}_{ij}$ is one entry (characterizing the ith and j^{th} beads) of $\underline{\boldsymbol{\sigma}}$ that is itself a 3×3 second-order tensor. $\vec{\boldsymbol{n}}_j$ is a uniformly distributed random number vector with entries between $[-1,\ 1]$. $\underline{\boldsymbol{D}}_{ij}$ represents the 3×3 second-order diffusion tensor that is one entry in the overall $3N \times 3N$ fourth-order diffusion tensor, $\underline{\boldsymbol{D}}$. The diffusion tensor, $\underline{\boldsymbol{D}}$, captures hydrodynamic interactions among bead members, such as shielding effects. $\underline{\boldsymbol{D}}_{ij}$ is approximated by the Rotne-Prager-Yamakawa tensor given by,

$$\underline{\boldsymbol{D}}_{ii}(\vec{\boldsymbol{R}}) = \frac{k_b T}{6\pi\eta a}\underline{\mathbb{I}}, \qquad i = j$$

$$\underline{\boldsymbol{D}}_{ij}(\vec{\boldsymbol{R}}) = \frac{k_b T}{8\pi\eta}\frac{1}{R}\begin{Bmatrix} \left(1 + \frac{2a^2}{3R^2}\right)\underline{\mathbb{I}} + \left(1 - \frac{2a^2}{R^2}\right)\frac{\vec{\boldsymbol{R}}\otimes\vec{\boldsymbol{R}}}{R^2}, & R \geq 2a \\ \frac{R}{2a}\left[\left(\frac{8}{3} - \frac{3R}{4a}\right)\underline{\mathbb{I}} + \frac{R}{4a}\frac{\vec{\boldsymbol{R}}\otimes\vec{\boldsymbol{R}}}{R^2}\right], & R < 2a \end{Bmatrix}, \quad i \neq j$$

where $\vec{\boldsymbol{R}} = \vec{\boldsymbol{r}}_i - \vec{\boldsymbol{r}}_j$ is the bead-bead separation vector with magnitude $R = \|\vec{\boldsymbol{R}}\|$, $\underline{\mathbb{I}}$ is the (3×3) identity tensor, and $a = 15\ nm$ is the bead radius.

Systematic Forces

The polymer chain has a globular affinity that is modeled using the 12-6 Lennard-Jones potential. $\vec{\boldsymbol{F}}_j^{LJ}$ is the resultant Lennard-Jones force, which is the sum of the $N-1$ pairwise additive Lennard-Jones forces between every bead pair in the ensemble involving the jth bead. $\vec{\boldsymbol{F}}_j^{LJ}$ is given by,

$$\vec{\boldsymbol{F}}_j^{LJ} = \frac{4\epsilon}{\sigma}\begin{Bmatrix} \left[12\left(\frac{\sigma}{R_j}\right)^{13} - 6\left(\frac{\sigma}{R_j}\right)^{7}\right]\widehat{\boldsymbol{R}}_j, & R_j \geq \frac{4}{5}R_{eq} \\ \left[12\left(\frac{\sigma}{\frac{4}{5}R_{eq}}\right)^{13} - 6\left(\frac{\sigma}{\frac{4}{5}R_{eq}}\right)^{7}\right]\widehat{\boldsymbol{R}}_j, & R_j < \frac{4}{5}R_{eq} \end{Bmatrix}.$$

where $\vec{\boldsymbol{R}}_j = \vec{\boldsymbol{r}}_i - \vec{\boldsymbol{r}}_j$ is the bead-bead separation vector with magnitude $R_j = \|\vec{\boldsymbol{R}}_j\|$, and $\widehat{\boldsymbol{R}}_j$ is the unit vector in the direction of $\vec{\boldsymbol{R}}_j$. $\epsilon = 0.5\ k_b T$ and $\sigma = (2a + 1nm)\ /\ \left(2^{1/6}\right)$ are the energy and

length parameters, respectively. To ensure numerical stability in the event of overlapping beads, $\vec{\boldsymbol{F}}_j^{LJ}$ is defined piecewise so that the force magnitude is truncated if beads overlap by more than approximately 80% of their radius. The separation distance demarking this truncation is given by $R_{eq} = 2a + 1nm$.

$\vec{\boldsymbol{F}}_j^{S}$ is the resultant spring force. Adjacent beads are connected by either finitely extensible nonlinear elastic (FENE) or harmonic springs that, respectively, take the form,

$$\vec{\boldsymbol{F}}_j^{FENE} = \frac{H\left(\vec{\boldsymbol{R}}_j - \vec{\boldsymbol{R}}_{eq}\right)}{1 - \left(\frac{R_j - R_{eq}}{R_{max}}\right)^2} \quad \text{and} \quad \vec{\boldsymbol{F}}_j^{Harm} = k\left(\vec{\boldsymbol{R}}_j - \vec{\boldsymbol{R}}_{eq}\right).$$

$\vec{\boldsymbol{R}}_j = \vec{\boldsymbol{r}}_i - \vec{\boldsymbol{r}}_j$ is the bead-bead separation vector with magnitude $R_j = \left\|\vec{\boldsymbol{R}}_j\right\|$. $\vec{\boldsymbol{R}}_{eq} = R_{eq}\widehat{\boldsymbol{R}}_j$ is the equilibrium spring length vector, where the equilibrium length is $R_{eq} = 2a + 1nm$ (measured from bead centers) and $\widehat{\boldsymbol{R}}_j$ is the unit vector in the direction of $\vec{\boldsymbol{R}}_j$. $H = 0.12\ pN/nm$ is the FENE spring constant and the maximum extensible length is $R_{max} = 51.5\ nm$, which were both obtained by fitting experimental A2 force-extension data[1]. $k = 100\frac{k_bT}{a^2}\frac{pN}{nm}$ is the stiff harmonic spring constant.

Simulation Information

Simulations began with vWF chains that were previously initialized in compact globular conformations at zero shear. In order to obtain sufficient statistics, a simulation ensemble consisted of 20 non-interacting chains. Each chain possessed a unique initial conformation and random seed number (for simulation). Both hydrodynamic interaction tensors were updated once every 1000 time steps (selection confirmed by convergence study). Feature and response information were collected once every 5,000 time steps. The time step employed was $\Delta t =$

$1.49\ ns$, and simulation durations were approximately 1.9×10^9 time steps or roughly $2.82\ sec$ total. vWF model and simulation parameters employed in this work are summarized in Table S1.

Coarse-Grained vWF Model Parameters			
vWF Monomer / Multimer	Bead Radius	Multimer Length	Harmonic Spring
	$a = 15\ [nm]$	$N = 100\ [Beads]$	$k = 100\ \frac{k_b T}{a}\ [pN/nm]$
FENE Spring	Equilibrium	Maximum	Constant
	$Q_{eq}^{FENE} = 1.0\ [nm]$	$Q_{max}^{FENE} = 51.5\ [nm]$	$H = 0.12\ [pN/nm]$
Lennard-Jones	Energy	Length	
	$\varepsilon = 0.5\ k_b T\ [pN\ nm]$	$\sigma = 1.0\ [nm]$	
Characteristic Quantities	Length	Time	Force
	$a = 15\ [nm]$	$6\pi\eta a^3 / k_b T\ [\mu s]$	$k_b T\ /\ a\ [pN]$
Parameters	Temperature	Viscosity	
	$T = 310\ [K]$	$\eta = 0.001\ pN\ \mu s\ nm^{-2}$	
**Coarse-Grained vWF Simulation Parameters**			
Simulation Duration	Time Step	Total Duration (22 Chains Total)	
	$\Delta\check{t} = 10^{-4} \cdot \frac{6\pi\eta a^3}{k_b T}\ [\mu s]$	$31.5\ [sec]$	
Data Collection	Data Output Frequency	Simulation Outputs Recorded	Total Outputs Used for RFA
	$5000\ \Delta\check{t}\ [\mu s]$	4,240,876	200,000

Table S1: Model and simulation parameters employed for coarse-grained molecular dynamics simulations, which generated data used as input to the random forest algorithm. All distances are measured from bead edges.

Prediction Importance

The two figures shown below, Fig. S1 and Fig. S2, illustrate the prediction importance distributions for the x-, y-, and z-direction position components within the various segments of the vWF multimer. Fig. S1 illustrates importance scores for the 10-segment resolution, and Fig. S2

illustrates the same for the chain-level resolution. Both figures clearly illustrate the prevalence of correlated segment dynamics, as discussed in the main text.

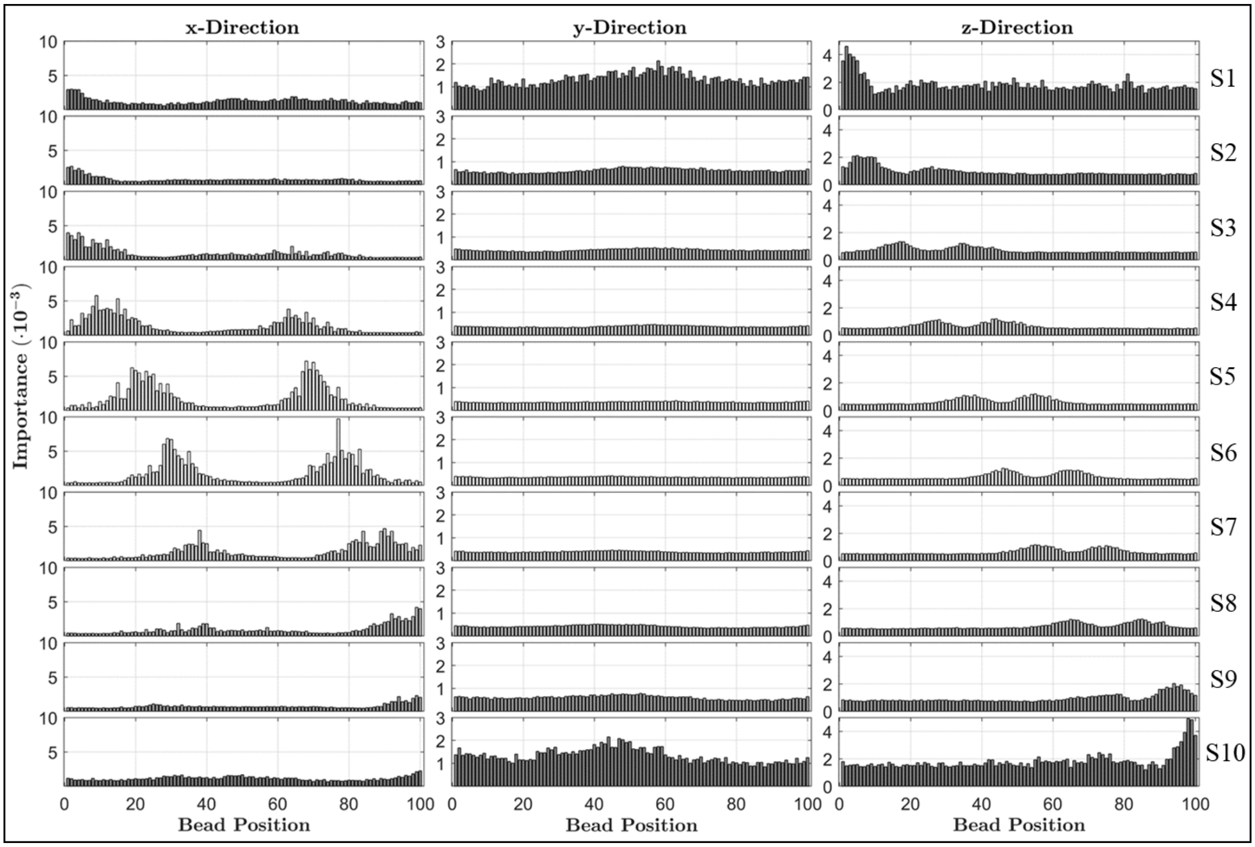

Figure S2: Distribution of x-, y-, and z-direction bead position importance scores along the multimer contour for the 10-segment partition resolution.

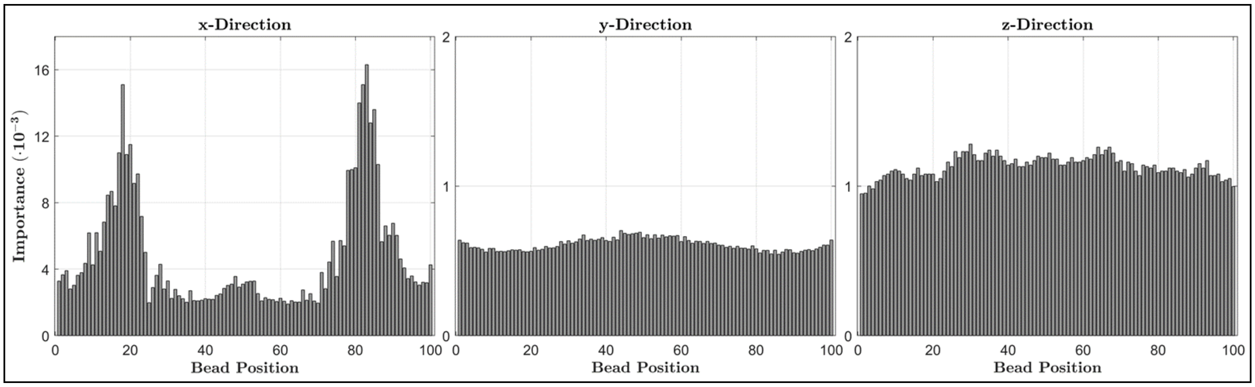

Figure S3: Distribution of x-, y-, and z-direction bead position importance scores along the multimer contour for the chain-level partition resolution.

Supplemental Material References:

1. Morabito, M. *et al.* Internal Tensile Force and A2 Domain Unfolding of von Willebrand Factor Multimers in Shear Flow. *Biophys. J.* (2018). doi:10.1016/J.BPJ.2018.09.001
2. Ouyang, W. *et al.* Flow-induced conformational change of von Willebrand Factor multimer: Results from a molecular mechanics informed model. *J. Nonnewton. Fluid Mech.* **217,** 58–67 (2015).
3. Wei, W. *et al.* Coarse-Grain Modeling of Shear-Induced Binding between von Willebrand Factor and Collagen. *Biophys. J.* **114,** 1816–1829 (2018).
